# Surgery of True Recurring Median Carpal Tunnel Syndrome with Synovial Flap by Wulle Plus Integument Enlargement Leads to a High Patient’s Satisfaction and Improved Functionality

**DOI:** 10.3390/jcm8122094

**Published:** 2019-12-01

**Authors:** Stephan Payr, Thomas M. Tiefenboeck, Veith Moser, Edvin Turkof

**Affiliations:** 1 Department of Orthopaedics and Trauma Surgery, Medical University of Vienna, 1180 Vienna, Austria; thomas.tiefenboeck@meduniwien.ac.at; 2 Department of Obstetrics and Gynecology, Medical University of Vienna, 1090 Vienna, Austria; vm@medspa.cc (V.M.); dr.edvin@turkof.com (E.T.)

**Keywords:** flap coverage by Wulle, integument enlargement, recurrent CTS, synovial flap

## Abstract

This prospective study was conducted to investigate electrophysiological qualities and patient’s satisfaction of a synovial gliding tissue flap in treating true recurring carpal tunnel syndrome. In 14 patients (11 women, three men), 15 median nerves were included in this retrospective study. For all 15 nerves, motor and sensory nerve conduction velocity, compound muscle action potential, a Visual Analogue Scale-score (VAS-score) questionnaire and an adapted Levine-Test were evaluated pre- and postoperatively. All participants underwent operative neurolysis of the median nerve, which was then enwrapped by a synovial gliding tissue flap. Eleven procedures were completed by integument enlargement. Follow-up period was 12 months. Postoperatively, distal latency decreased significantly by 15.6%. Compound muscle action potential and sensory nerve conduction velocity did not improve significantly. VAS score regarding pain reduced highly significantly with 74.1%. The adapted Levine-Test function score improved highly significantly with 39.2%. The synovial gliding tissue flap lead to an excellent patient’s satisfaction for treating true recurring carpal tunnel syndrome. Primary wound closure should be completed with integument enlargement if needed.

## 1. Introduction

In the literature, large series of nerve releases to treat median carpal tunnel syndrome (CTS) show a complication rate [1,2,3,4,5,6,7,8,9,10,11,12,13,14,15,16] (treatment failure) that varies from 3 to 25% and a reoperating rate of 12% [3,7,8,9,10,11,17]. Additionally, multiple neurolysis (external or internal) alone is rarely the appropriate treatment for true recurring carpal tunnel syndrome, because the re-operated nerve is often trapped in fibrous scar tissue resulting from the repeated disintegration of the surrounding gliding tissue [1,7,18,19,20,21]. Therefore, most authors complete secondary neurolysis with any type of flap coverage of the median nerve [1,7,11,15,17,22,23,24,25].

The idea of embedding the median nerve into a well-vascularised tissue in order to prevent recurrent scarring was first introduced by Brown and Flynn with an abdominal pedicle flap in 1973 [26]. Subsequently, several similar procedures were developed, with the hypothenar fat pad flap (HTFPF) and the synovial flap coverage by Wulle being two of them [3,22,23,25,27,28,29,30,31]. According to some studies, the synovial flap method seems to produce inferior results compared to the more frequently applied HTFPF method [3,12,15,17]. However, unlike the HTFPF, relatively little has been published about the synovial flap, and we could find only one study, which directly compares these two techniques. In contrast, we regard the synovial flap coverage by Wulle to be a viable procedure, which produces highly satisfactory results [3,22,31]. Therefore, we conducted this prospective study to investigate electrophysiological qualities and patient’s satisfaction of the flap coverage by Wulle in the treatment of true recurring CTS according to the Wulle’s classification.

## 2. Material and Methods

Ethical approval was granted for this study by the Ethics and Scientific Review Committee of the Medical University of Vienna (1722/2016) and informed consent was acquired from all participants.

All patients were operated on by the same surgeon (ET) and all electrophysiological tests were performed by the same investigator (VM) as well as the subjective assessment by SP.

### 2.1. Patient Selection

The inclusion criteria were based on those characteristics, as defined by Wulle, which constitute true recurring CTS [22]. Accordingly, the following points led to exclusion from the study: symptom-free interval of less than 3 months between first surgery and revision surgery, incomplete transection of the transverse carpal ligament at the primary intervention, iatrogenic median nerve laceration and a double crush syndrome based on clinical examination. Furthermore, patients suffering from any kind of systemic neuropathy if present in the patient’s medical history, as well as any rheumatic or vascular disease, were also excluded in order to further increase the homogeneity of the study population. Fourteen patients (11 women and three men) aged between 22 and 77 years (mean age: 48.5 ± 13.18 years) were included in the study. Both hands of one patient were unsuccessfully operated upon before the study. Two patients had been previously operated upon twice—and one patient three times—before they were treated by us (see Table 1).

In all cases, patients’ histories showed a symptom-free interval of four to fourteen months (mean: 8.5 ± 2.9 months) and persistent sensibility disorders, as well as intolerable constant pain after the relapse of the symptoms. Consequently, all patients included in this study were considered as suffering from true recurring CTS [3]. The time interval between the first/last operation and revision surgery ranged from 13 to 29 months (mean: 20.3 ± 4.8 months). Each patient had tried conservative pain treatment with splints and NSAID medications for several months prior to revision surgery.

### 2.2. Pre- and Postoperative Investigations

Patients underwent electrophysiological testing and clinical subjective assessment (VAS-score and Levine test function-score).

Clinical evaluation included the presence of Tinel’s sign, Phalen and reversed Phalen sign, the presence of paraesthesia in the peripheral area of the median nerve and the presence of night pain. Night pain is referred to as any condition including pain, tingling sensation, numbness and paraesthesia during the night. In general, any sensation that leads to discomfort and waking up during night-time interrupting patient’s sleep but typically improves when shaking the hand is performed.

VAS-score from 0 to 10 was used to determine each patient’s level of perceived pain (0 = no pain, 10 = extreme pain), limitation of activities (0 = no problems, 10 = severely limited in performing daily tasks) and satisfaction with the results of their surgery (0 = completely dissatisfied, 10 = extremely satisfied). Satisfaction with the results of surgery was evaluated only postoperatively. To verify and strengthen results collected by the VAS-score test, a Levine test function-score was additionally performed to assess subjective outcomes for pain relief and function [32]. All 14 patients were evaluated by the same observer (SP).

Electrophysiological testing was performed with a Dantec-Keypoint electromyograph and its built-in software. Testing consisted of measuring the motor distal latency (abductor pollicis brevis muscle, 6.5 cm-Ludin, surface electrode, rectangular stimulating impulse, 0.1 msec stimulus duration) and the sensory nerve conduction velocity (NCV) (ring-electrodes, digit II, antidromic, onset latency, 10 × averaging). Care was taken to maintain standardized conditions (same room, similar room temperature, skin temperature not below 34 °C). Electromyography was not performed, nor was inching. All electrophysiological tests were performed pre- and postoperatively by the same experienced electrophysiological investigator (VM).

For statistical analysis, preoperative test were administered one week before surgery and the results from the last postoperative testing interval (12 months after surgery) were used.

### 2.3. Operative Technique

**Figure 1:**
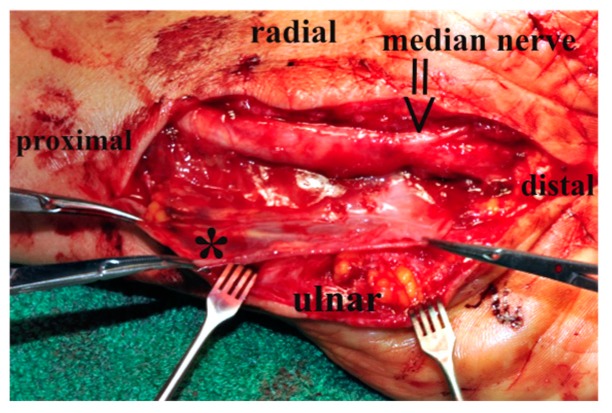
Operative site prior to wrapping the median nerve with the synovial flap; view of the median nerve (≥) after neurolysis. * The synovial bag of the flexor tendons.

All interventions were performed by the same qualified hand surgeon experienced in the synovial-flap by Wulle using a standardized technique (ET). A tourniquet was placed around the upper arm and inflated to 250–300 mmHg. Skin incision was performed along the pre-existing scar, which ran in all 15 cases in a curved line along the linea vitalis and crossed the volar crease of the distal forearm. Subsequently, the subcutaneous tissue was dissected and the tendon of the palmaris longus muscle was transected to remove 4–6 cm of its distal portion, in order to prevent adhesions between the nerve and the tendon. The median nerve was then identified and hooked with a vessel loop. After completion of exposing the nerve, pictures of the nerve were taken Figure 1 and the tourniquet released. If the exposed nerve appeared to be constricted and/or flattened, and would not expand subsequent to release of the tourniquet, indication for internal neurolysis was declared and epineurotomy performed.

**Figure 2:**
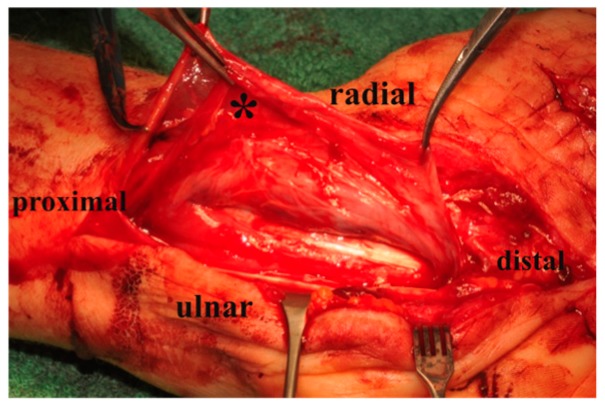
Harvesting the flap from the common synovial bag of flexor tendons (*); view of exposed flexor tendons from ulnar.

**Figure 3:**
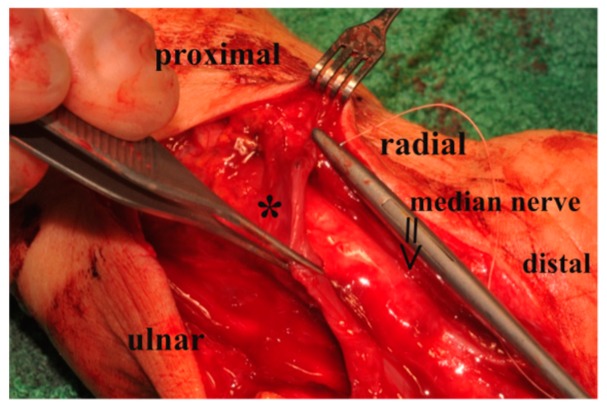
Operative site of wrapping the median nerve (≥) with the synovial flap (*) from ulnar to radial.

**Figure 4:**
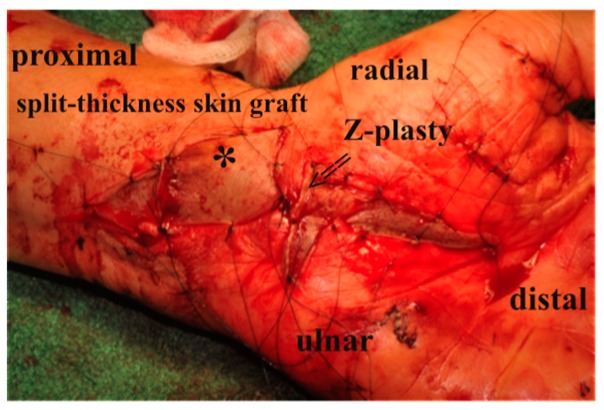
Wound closure with split-thickness skin graft (*) over the synovial gliding tissue flap and a Z-plasty (≥) at the wrist to provide integument enlargement as primary wound closure would exert too much pressure on the median nerve.

Next, the gliding tissue flap was elevated from the common synovial bag of flexor tendons and wrapped around the nerve Figure 2 and Figure 3. In order not to exert too much pressure on the nerve, integument-enlargement was performed. Therefore, wound edges at the flexor fold were adapted with a Z-plasty to achieve a transverse scar, and the exposed flap was covered distally and proximally to the Z-plasty by split-thickness skin graft Figure 4. Postoperatively, a splint was given for 6 days. After this period, motion until pain was possible, full motion was restored after 12–14 days.

### 2.4. Statistical Analysis

The pre-and 12 months postoperative NCV, VAS-score and Levine test function-score values in the two groups were compared with the Student’s *t*-test for paired data. Descriptive data (mean ± SD) are reported for the entire patient cohort. The level of significance was set at a *p*-value of ≤ 0.05. Statistical analysis was performed using Microsoft Excel^®^, SPSS^®^ software (Version 22.0, SPSS Inc.: Chicago, IL, USA).

## 3. Results

In surgery, all 15 nerves were found to be surrounded by extensive fibrous tissue within the carpal canal; therefore, all 15 nerves were covered by a synovial gliding tissue flap subsequent to neurolysis. Epineurotomy and internal neurolysis were performed in nerve number 2 and 9, since these two nerves remained constricted after release of the tourniquet. In 11 out of 15 hands, an integument enlargement was performed. All 15 hands showed a significant improvement regarding pain (see VAS score and Levine Test) with additional improvement in sensation (patient statements). Tinel’s sign were present preoperatively in all 15 hands and remained but weakened in all cases. Night pain, including tingling sensations and paraesthesia, was present preoperatively and disappeared postoperatively in all hands with additional improvement of functionality evaluated by VAS. The results of pre- and postoperative tests showed a postoperative improvement in the majority of patients and are listed in Table 2.

**Figure 5:**
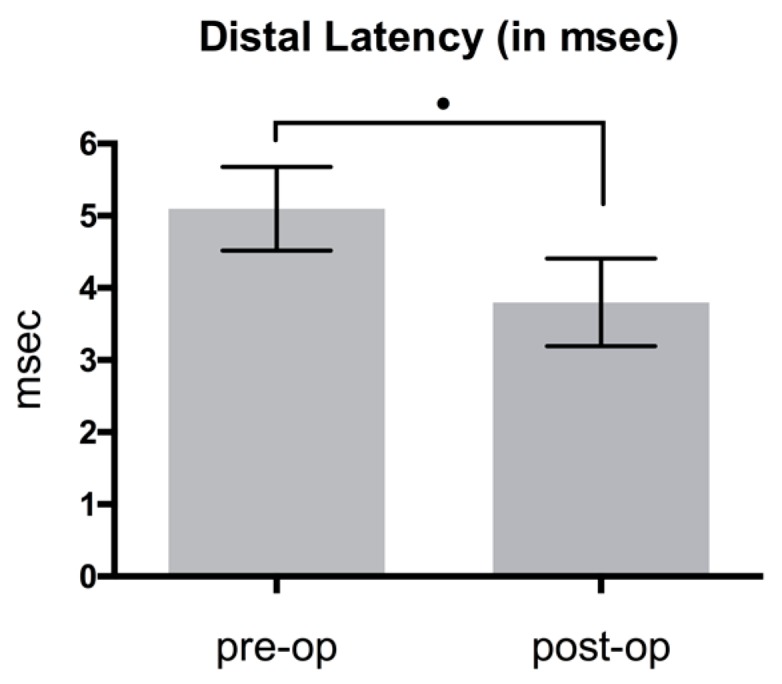
Comparison of pre- and postoperative distal latency (*p* < 0.05).

**Figure 6:**
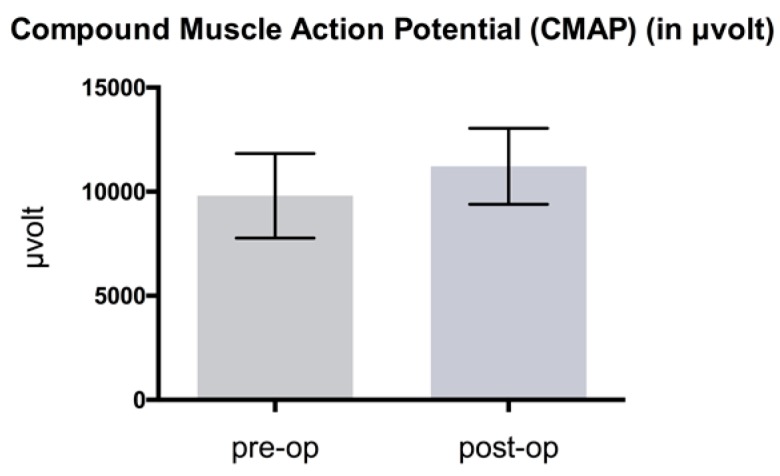
Comparison of pre- and postoperative compound muscle amplitude potential in µvolts (*p* > 0.05).

**Figure 7:**
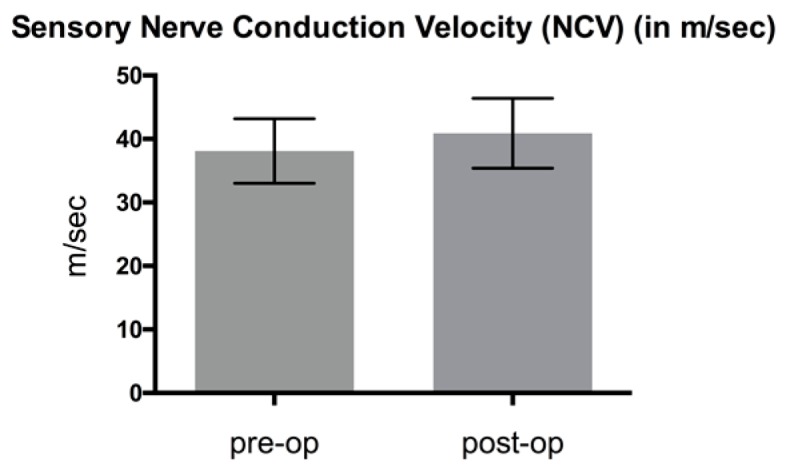
Comparison of pre- and postoperative sensory nerve conduction velocity (*p* > 0.05).

NCV showed an improvement when comparing mean pre- and postoperative results as shown in Figure 5, Figure 6 and Figure 7.

**Figure 8:**
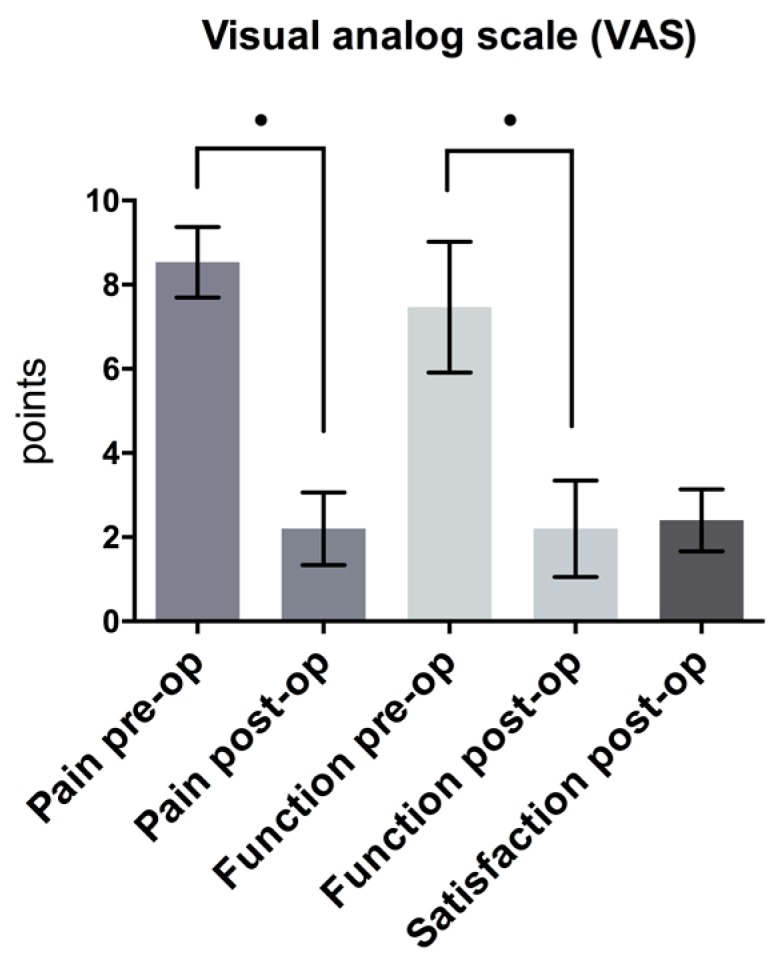
Comparison of pre- and postoperative VAS Score (pain and function) (*p* < 0.001) and satisfaction postoperatively.

Distal latency decreased 25.5%, from 5.1 ± 0.58 to 3.8 ± 0.61 ms; difference: 1.3 ms (*p* < 0.05) (Figure 5). Compound muscle action potential (CMAP) improved by 14.4% from mean 9802.9 ± 2028.55 to 11214.3 ± 1825.01 μV; difference: 1411.4 μV (*p* > 0.05) (Figure 6). Sensory NCV values increased postoperatively from 38.1 ± 5.08 to 40.9 ± 5.5 m/sec; difference: 2.8 m/sec (*p* > 0.05) (Figure 7). The VAS-score regarding pain showed an highly significant reduction of 74.1%, from 8.5 ± 0.8 to 2.2 ± 0.9 points; difference: 6.3 points (*p* < 0.001) (Figure 8). The VAS-score regarding function revealed a significant improvement of 7.5 ± 1.6 to 2.2 ± 1.2 points; difference: 5.3 points (*p* < 0.001). The overall postoperative satisfaction was 2.4 ± 0.7 points. This represents a highly significant reduction in pain and a high patient’s satisfaction with the result after surgery.

**Figure 9:**
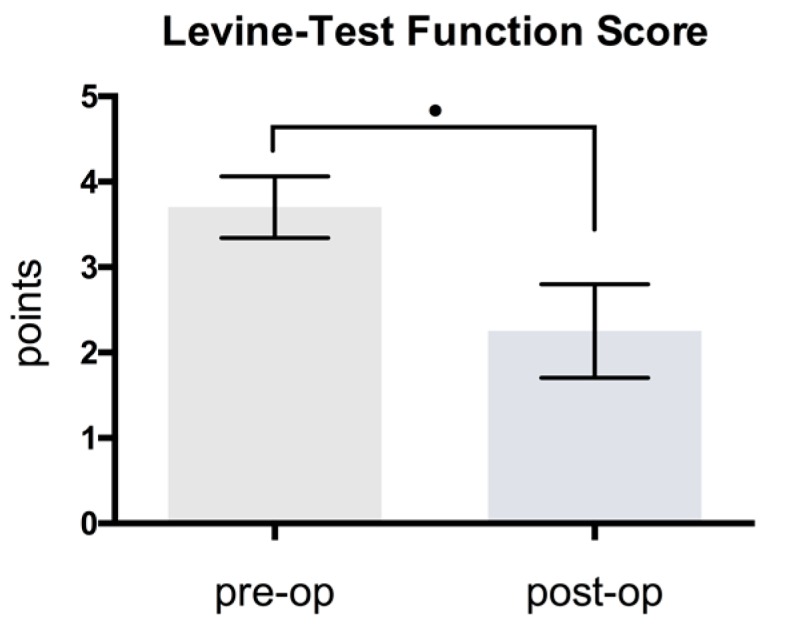
Comparison of pre- and postoperative Levine Test Function Score (*p* < 0.001).

The Levine test function-score also indicated a highly significant improvement of 39.2%, from 3.7 ± 0.36 to 2.25 ± 0.55 points; difference: 1.45 points (*p* < 0.001) (Figure 9). This underlines the explicit subjective improvement of pain and function. Distal latency exhibited a clinically relevant improvement of at least 20%, except in patients 5, 9 and 14. A comparison of the pre- and postoperative mean values indicated a statistically significant improvement (*p* < 0.001). The CMAP and sensory NCV did not lead to statistically significant results (*p* > 0.05), whereas the VAS-score and the Levine test function-score presented highly significant results (*p* < 0.001) (Table 3).

Astonishingly, the split thickness skin graft was never a subject of complaint by any patient, and accordingly, revision of the site was never requested.

## 4. Discussion

In the literature, several papers contend that the gliding tissue flap is considered not to be the ideal method to treat recurring CTS, as it provides insufficient padding and inadequate size [1,3,12]. One author refuses to employ the flap, claiming its tendency to undergo fibrosis and to form scar tissue [33]. In our hands, however, the synovial gliding tissue flap proved itself to be an excellent method of treating recurring CTS—provided that wound closure is not performed under pressure [34].

True recurring CTS is caused by median nerve fibrosis. The fibrosed nerve is unable to glide forward and backward to follow the movement of the wrist—thus causing pain as the nerve is continually stretched and compressed [22]. Consequently, the paramount goal of treating recurring CTS—in addition to performing neurolysis and decompression of the median nerve—has to be the avoidance of postoperative recurring fibrosis. The best way to prevent fibrosis is to refrain from placing the nerve into a fibrotic wound bed and to avoid wound closure under pressure [22,31,34]. Avoiding a fibrotic wound bed can be achieved by wrapping the nerve in a well-vascularised tissue [22,31]. The synovial gliding tissue flap is very well-vascularised, envelops the median nerve completely and moreover provides the mechanical properties of a lubricant. Regarding wound closure under pressure, Millesi states that it is preferable to perform an integument enlargement—instead of a primary wound closure—if the wound status suggests that the primary closure would leave the wound under pressure [34].

In the literature, the most popular flap for treating recurring CTS appears to be the HTFPF, with authors hailing its excellent padding properties [1,3]. However, a constricted median nerve does not need protection against pressure, because the exertion of pressure upon nerves must be avoided during surgery anyway [34]. Furthermore, authors noticed that postoperative swelling more often and more strongly occurred after surgery of recurring CTS when compared with interventions of primary cases; therefore, primary wound closure after surgery of recurring CTS might often lead to increased pressure on the median nerve. Consequently—especially in recurring cases—one has to be aware of the amount of pressure necessary to close the wound.

The HTFPF is definitively thicker than the synovial gliding tissue flap. Thus, the HTFPF can better protect a re-exposed median nerve against too much pressure due to wound closure; with respect to its ability to offer mechanical protection, the synovial flap is too thin to serve such a purpose. However, a suitable flap should in the first place provide for good gliding of the median nerve—not particularly for shock absorption. This opinion has been shared previously by other authors, who claim that the small bulk of tissue provided by the synovial gliding tissue flap is just sufficient enough mechanical protection [11,31]. Moreover, when using the HTFPF, the intra-canal volume increases due to its fatty tissue [11,31].

Thus, the quoted padding of the nerve seems to be unnecessary, because wound closure should be performed in a way to strictly avoid any pressure on the median nerve [34]. This can be achieved—if necessary—by enlarging the integument with a split thickness skin graft placed just on the flap. These considerations render citations regarding the inferior padding capability of the synovial gliding tissue flap irrelevant.

Another mentioned inconvenience of the synovial gliding tissue flap found in the literature is the flap’s supposed inadequate size [1,3,12]. According to our personal experience over the last 20 years of surgery and several hundred CTS-interventions, the observed area of fibrosis of the median nerve within the carpal canal showed a variable length of approximately 1.5–5.5 cm. Given the fact that the length of the synovial bag is much longer, this cited inconvenience has not occurred in our hands.

Finally, it can be questioned if not only one surgical step described in the operative technique might have led to the same results—with less effort. However, we believe that we had to stay close to our ethical guidelines, and completion of a surgical treatment without implementing all necessary steps is unacceptable. Leaving a fibrotic nerve unprotected and closing a wound under pressure is considered to be wrong; therefore, we performed the complete procedure with every step necessary.

Furthermore, the HTFPF is less applicable when compared to the synovial gliding tissue flap for two reasons. Firstly, due to the restricted length of both the flap and its pedicle, the HTFPF cannot cover lesions longer than 2 cm [11]. Secondly, the HTFPF cannot be used if the lesion is located too proximally—i.e., at the distal part of the antebrachial fascia [11]. Given this, we regard the synovial tissue flap to be suitable in all cases of recurring CTS—which cannot be said for the HTFPF.

Our clinical results underline the suitability of the flap, and it is not contradictory that only two patients regained normative values of their distal latency, as NCV studies often reveal residual slowing of distal sensory latency even after a successful carpal tunnel surgery, especially in chronic cases [35]. Therefore, we find the subjective patient’s satisfaction 12 months after the surgical procedure to be far more representative than the electrophysiological findings.

The synovial flap exhibits other, rather convenient properties for the treatment of recurring CTS:

* The synovial flap is the only flap capable of fully enwrapping the exposed median nerve, thereby effectively protecting the nerve against sprouting fibrotic tissue.
* Donor site morbidity is minimal. This leads to a satisfactory cosmetic outcome, especially when primary wound closure is possible.
* Dissection of the synovial flap is quick and simple, the learning curve short and the complication rate very low (based on personal observation).

In our hands, the synovial gliding tissue flap proved to be an excellent tool in treating true recurring CTS. To avoid pressure on the wound, and therefore compressing the nerve again, best results are obtained with an integument enlargement.

## Figures and Tables

**Table 1:** Patient overview. Patients’ details are listed with the number of previous interventions, symptom free interval and elapsed time between the last intervention and our synovial flap plasty. Patient 5 had undergone three previous surgeries on the right dominant hand, and patient 7 had both hands previously operated upon.

Patient (*n*)	Age (Years)	Gender (F/M)	Side (l/r) Dominant Hand (+/−)	Number of Previous Interventions	Symptom-FreeInterval (Months)	Elapsed Time between Last Intervention and the Synovial Flap Plasty (Months)
1	32	F	r +	1	8	16
2	77	F	r +	1	6	17
3	56	M	l +	2	5	14
4	64	M	r −	1	10	27
5	46	F	r +	3	4	15
6	22	F	l +	1	5	13
7	51	F	l −/r +	1	9/6	18/24
8	62	F	l +	2	7	18
9	48	M	r +	1	12	26
10	43	F	r −	1	14	29
11	42	F	r +	1	11	20
12	51	F	r −	1	13	24
13	41	F	l +	1	8	20
14	44	F	r +	1	9	23

**Table 2:** Nerves’ pre- and postoperative results. 7 and 8 are nerves from the patient who had both hands operated upon.

Nerve	Primary Wound Closure (Yes/−)	Integument Enlargement (Yes/−)	Motor Distal Latency (msec) Pre/Post	Normative Values of Distal Latency	CMAP (μvolt) Pre/Post	Sensory NCV (m/sec) Pre/Post	VAS Pain Pre/Post	VAS Limitations of Activities Pre/Post	Levine Pre/Post
1	Yes	−	4.5/3.6	2.8	11849/12833	43/46	9/2	7/2	3.4/1.9
2	−	Yes	5.7/4.4	3.0	8927/9596	37/41	9/2	9/2	3.8/2.7
3	−	Yes	4.4/3.5	3.0	7756/9014	33/35	10/4	10/4	4.0/2.8
4	−	Yes	5.0/3.8	3.1	10679/13415	40/40	9/2	7/2	3.6/2.0
5	−	Yes	6.7/5.4	2.8	5483/7797	28/30	10/3	10/5	4.4/3.3
6	Yes	−	4.9/3.0	2.8	14123/14632	48/54	8/3	6/3	3.0/1.1
7		Yes	4.7/3.6	2.8	11619/12110	38/41	8/2	6/2	3.9/2.3
8	−	Yes	4.9/3.6	2.8	9013/10318	39/43	8/2	8/2	3.5/2.1
9	−	Yes	5.6/4.6	2.9	7987/9583	31/35	8/2	8/2	3.3/2.4
10	−	Yes	4.6/3.3	3.2	9365/12846	36/39	9/3	5/1	3.8/2.9
11	−	Yes	5.3/3.6	2.8	9190/10129	45/47	8/1	8/1	4.1/3.2
12	Yes	−	4.9/3.8	2.8	10241/12299	40/43	8/3	6/3	3.2/2.4
13	−	Yes	5.0/3.8	2.8	8421/9958	41/42	9/1	9/2	3.9/2.5
14	Yes	−	5.6/4.7	2.8	10593/11727	39/41	7/1	7/3	3.6/2.9
15		Yes	4.7/3.4	2.8	11797/11958	34/37	8/1	6/1	4.0/3.0

**Table 3:** Pre- and postoperative mean values. * Significant results.

	Pre-op [MV]	Post-op [MV]	Significance
Distal Latency *	5.1	3.8	*p* < 0.05
CMAP	9802.9	11214.3	*p* > 0.05
Sensory NCV	38.1	40.9	*p* > 0.05
VAS Pain *	8.5	2.2	*p* < 0.001
VAS Function *	7.5	2.2	*p* < 0.001
VAS Satisfaction	−	2.4	
Levine *	3.7	2.25	*p* < 0.001
